# Hemp Shives as a Raw Material for the Production of Particleboards

**DOI:** 10.3390/polym14235308

**Published:** 2022-12-05

**Authors:** Radosław Auriga, Marta Pędzik, Robert Mrozowski, Tomasz Rogoziński

**Affiliations:** 1 Department of Mechanical Wood Processing, Institute of Wood Sciences and Furniture, Warsaw University of Life Sciences—SGGW, 02-776 Warsaw, Poland; 2 Center of Wood Technology, Łukasiewicz Research Network, Poznań Institute of Technology, 60-654 Poznań, Poland; 3 Department of Furniture Design, Faculty of Forestry and Wood Technology, Poznań University of Life Sciences, 60-627 Poznań, Poland

**Keywords:** wood-based materials properties, three-layer particleboard, alternative raw material, non-wood

## Abstract

Increased demand for wood affects its price and thus contributes to the growing interest in raw materials that can be used as a partial or total substitute for wood in the production of particleboard. One of the raw materials for the production of particleboard can be Cannabis sativa or, more precisely, hemp shives. In this work, 7 variants of panels with a density of 650 kg/m^3^ with 10 and 25% hemp shives substitution in different layers were produced. Particleboards containing hemp shives were characterized by lower density compared to conventional particleboards. The shares of hemp shives at the levels of 10% and 25% have a slight impact on the MOR and MOE; additional IB showed no statistically significant differences between the conventional particleboards and particleboards with a share of hemp shives. For particleboards with 25% hemp shives, a reduction in swelling was observed relative to particleboards made entirely of industrial wood particles.

## 1. Introduction

The global production of wood-based materials has been showing a constant upward trend for years. Among the wide range of wood-based materials available on the market, particleboard occupies a special place. The production of particleboard in the world in 2020 alone amounted to 102 million cubic meters [1]. Along with the increase in the volume of particleboard production, the demand for the raw material from which this type of material is produced increases. The main material for production is wood. Limited availability and an increased demand for wood indicate its price and exacerbate competition over declining resources [2]. Thus, they are contributing to the growing interest in raw materials that can be used as a partial or complete substitute for wood. Some types of lignocellulosic raw materials, including non-wood raw materials, can cover the demand of manufacturers of wood-based panels to a small extent, which forces their combination [3]. These include willows (*Salix vimnalis* L.) [4], giant miscanthus (*Miscanthus giganteus*) [5,6], grasses [7] and straws [8,9].

Recently, in terms of use in the particleboard industry, a lot of attention has also been paid to lignocellulose agricultural waste [10,11]. Research on agricultural waste biomass concerned the application of corn cobs [12,13], sunflower husk [14], hazelnut and walnut shells [15,16], brewer’s spent grain [17] and apple wood [18] or plum wood [19,20]. The disadvantage of agricultural waste lignocellulosic materials is that they often have additional substances, such as waxes that may adversely affect the gluing process with conventional resins, and thus reduce the mechanical properties of the manufactured boards [21,22].

One of the promising raw materials for the production of particleboard is Cannabis sativa. According to Moulana [23], the development of hemp-based composites is an interesting alternative to protect the forests and the problems associated with the shortage of wood raw materials. Cannabis sativa is a universal plant that is quite easy and environmentally friendly to grow. Currently, industrial hemp is grown primarily to obtain seeds for the production of oils and other nutritional supplements, which are very popular [24,25]. Additionally, hemp fiber is extremely strong and durable, which is why it is used in the production of ropes, fabrics, paper, fiberboards and insulation boards. The tensile strengths and tensile modulus for hemp fibers at a fiber diameter of 4 μm are about 4200 and 180 GPa, respectively, and decrease with larger fiber diameter; e.g., for a 66 μm diameter fiber, they are 250 MPa and 11 GPa, respectively [26]. These values are lower than those of glass fibers but nevertheless good enough to be used as reinforcement in composite materials. In addition, these fibers can be used successfully as reinforcement for building materials based on cement, hydraulic lime and gypsum binders [27,28,29]. Such numerous uses result in a significant amount of residue, which, when properly prepared, can be used for other purposes.

Decortication of the stalks leads to three main fractions: long fibers, short fibers and woody core tissue. The woody core part of the hemp stalk, which is called hurd (or shives when in the form of industrial particles), is considered waste in the hemp industry. Nevertheless, hemp shives are used as an energy raw material, as well as a raw material for the production of various types of composites. There were also several works on the use of hemp shives as a raw material for the production of particleboards [23,30]. The results obtained by Schopper et al. [31] for the production of hemp-based three-layer particleboard using hemp hulls and leaves indicate that it is possible to produce lightweight particleboard (450 kg/m^3^ and 550 kg/m^3^) using hemp. They also pointed out that there are problems with their use as a raw material for particleboard production, as the boards do not meet all the requirements of the European standard in terms of the mechanical and technological properties of the boards, such as hemp particles being too fine after shredding, which causes them to absorb a large amount of adhesive. The study by Zvirgzds et al. [32], on the other hand, describes the properties of particleboards made with hemp particles in two size ranges of 0.5 mm to 2 mm and 2 mm to 5.6 mm as filler. Moulana [23] states that hemp shives can be successfully used in the production of particleboard as a substitute for wood in the core layers of the manufactured boards. The produced 550 kg/m^3^ boards with a hemp core inner layer and wood surface layers had high internal bonding strength (0.68 N/mm^2^) but low bending strength 11.8 N/mm^2^. In the production of particleboards, for technological reasons, differentiating the raw material composition of particles into core and face layers is quite a difficult procedure, especially when the material used is brittle and not homogeneous. In addition, the much lower bulk density of hemp shavings relative to wood shavings can translate into problems with the size of the pressing mat and the need to reduce the density of the boards and can, consequently, lower the performance of the finished boards. Research perspectives in this area should focus even more deeply on combining environmental, economic and social aspects of sustainability through eco-friendly and recyclable particleboards [2]. Both the demand for quality materials and the need for more environmentally friendly technologies are increasing [33]. Therefore, as part of the research, it was decided to investigate the effect of industrial hemp as a substitute for wood raw material in individual layers of the manufactured boards on the mechanical and physical properties of particleboards.

## 2. Materials and Methods

### 2.1. Materials

For manufacturing the particleboards industrial particles, mainly softwood and post-consumer wood were used. The wood industrial and hemp shives particles were characterized by an absolute humidity of 8%, both for the surface layers and the core layer.

The fractional compositions of the industrial particles and hemp shives used for the core and surface layers are shown in Table 1. Determination of the fractional composition of the raw materials was carried out on a laboratory vibrating screen AS 200 tap (Fritsch, Idar-Oberstein, Germany) with the following mesh sieves: 6.0, 4.0, 2.0, 1.25, 0.63, 0.49, 0.385 and <0.385 mm. The core layer particles passed through the 4 mm mesh and were retained by the 2 mm mesh. The particles used for the surface layer passed through the 2 mm mesh and were retained by the 0.25 mm mesh.

**Figure 1:**
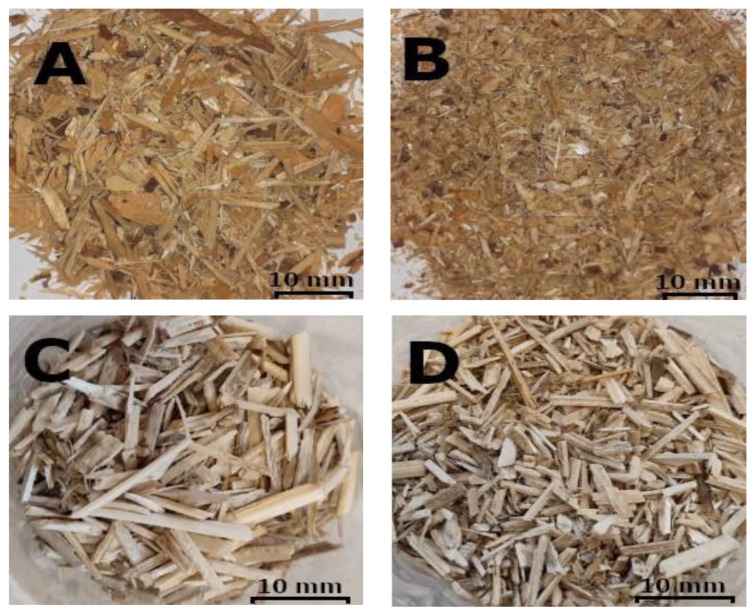
Particle size for each layer of the board: (**A**) industrial particles for the core layer, (**B**) industrial particles for the surface layers, (**C**) hemp shives for the core layer, (**D**) hemp shives for surface layers.

Figure 1 shows the particle sizes of the two raw materials for each layer of the panels. The reason for the selection of the indicated sizes of vortices used in individual layers was the use of particle mixtures dimensionally similar to the dimensions of industrial particles used in the production of particleboards.

In the experiment, the surface layers and core layers were sealed with the same resin and using the same adhesive formulation. The adhesive used in the experiment was based on urea-formaldehyde resin (Silekol 120 by the factory Silekol Sp, z o.o. (Kędzierzyn-Koźle, Poland)) with a dry matter content of 67%, a relative density of 1.30 g/cm^3^ and a dynamic viscosity of about 500 mPa∙s. The hardener was a 10% aqueous solution of ammonium sulfate. The unit composition of the adhesive was as follows: 50:15.5:1.5 UF resin, water and hardener, respectively. The amount of hardener was selected to achieve an adequate gel time (120 s) to ensure adequate curing of the adhesive resin when the panel was pressed.

### 2.2. Particleboards Manufacturing

As part of the research, three-layer particleboards with a density of 650 kg/m^3^ and a mass fraction of hemp shives at the levels of 0%, 10% and 25% were used. The hemp shives were added to the core and/or surface layers (Table 2). The assumed thickness of the panels was 16 mm, the degree of gluing of the surface layers 10% and the core layer 8%. The share of face layers in the board was 35%.

The pressing process was carried out in hydraulic press PH-1LP25 (ZUP-NYSA, Nysa, Poland) using standard characteristics of particleboard technology conditions (unit pressure of 2.5 MPa, temperature of 180 °C and pressing factor of 20 s per one mm of nominal board thickness) [34,35]. Manufactured particleboard was conditioned at a temperature of 20 ± 2 °C and relative air humidity of 65 ± 5% for at least 7 days.

### 2.3. Experimental

The modulus of rupture (MOR) and modulus of elasticity (MOE) were determined according to European Committee for Standardization EN 310:1994 [36]. Internal bonding was determined through the tensile test perpendicular to the surface of the board, according to EN 319:1999 [37]. All mechanical tests were conducted via a laboratory testing machine custom-made by Research & Development Center for Wood-Based Panels Sp. z.o.o. in Czarna Woda, Poland. At least 10 samples were used to determine each property.

The density of manufactured boards was determined according to the EN 323:1999 standard [38]. Additionally, the density profile was assayed in three replications. The 50 × 50 mm samples were analyzed via a GreCon Da-X (X-ray) measuring instrument (Fagus-GreCon Greten GmbH & Co. KG, Alfeld, Germany) with the incremental step of 0.02 mm/s.

Thickness swelling (TS) and water absorption (WA) after 2 h and 24 h of soaking in water were measured according to EN 317:1999 [39].

### 2.4. Statistical Analysis

Statistical analysis of the obtained results was carried out on Statistica 13 (TIBCO Software Inc. (2017). Analysis of variance (ANOVA) was used to test (α = 0.05) for significant differences between factors. A comparison of the means was performed by the Tukey test, with a 0.05 significance level.

## 3. Results and Discussion

**Figure 2:**
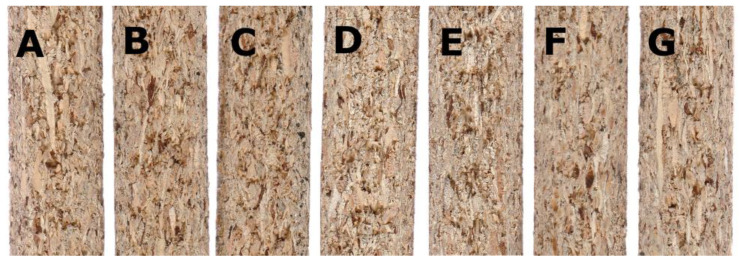
Cross-section of the manufactured particleboard variants. (**A**–**G**) letters designations according to the variant letters in Table 2.

The particleboards manufactured as part of the research were characterized by average density in the range of 634–651 kg/m^3^ (Table 3). It should be noted that the difference in the density of the manufactured boards in comparison to the assumed density (650 kg/m^3^) did not exceed 4%. Moreover, the difference in the densities of the boards produced within the individual variants was not statistically significant. Figure 2 shows cross-sections of all manufactured board variants.

**Figure 3:**
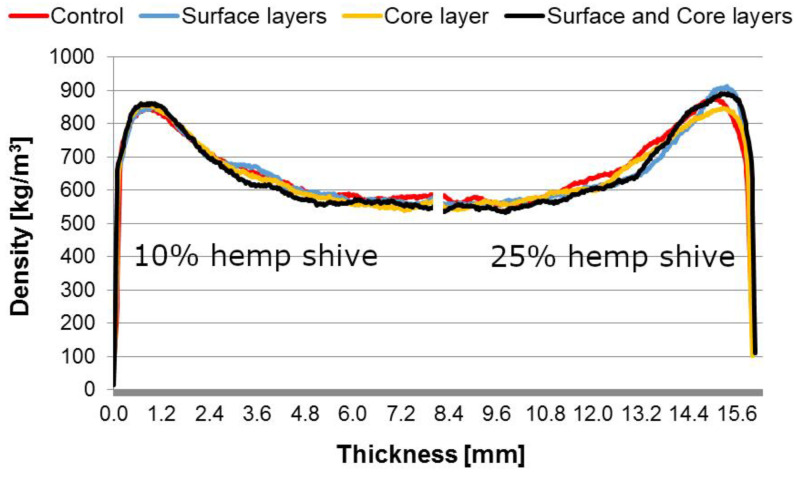
Density profile of manufactured particleboards.

All manufactured particleboards were characterized by a shaped vertical density profile typical for this type of wood material. Particleboards made with a 10% share of hemp shives showed no significant differences in the density of the face layer and core layer in comparison to boards made entirely of industrial wood particles (Figure 3).

In the case of particleboards with 25% hemp shives, slight differences were observed in the density profile in comparison to boards made entirely of industrial particles. Particleboards with a 25% share of hemp shives in the core layer were characterized by the slightly lower density of the surface layers compared to particleboard made from wood industrial particles, while in the case of particleboards with 25% hemp shives, in regard to face layers, a higher density of the face layers was observed. Particleboards containing hemp shives were characterized by lower density compared to conventional particleboards. The lower density as well as the differences in the compaction of the manufactured particleboard with hemp shives may result from the much lower bulk density of hemp shives compared to wood. It is worth noting that both the density and the density profile correlate with the mechanical properties of the particleboards [40,41].

**Figure 4:**
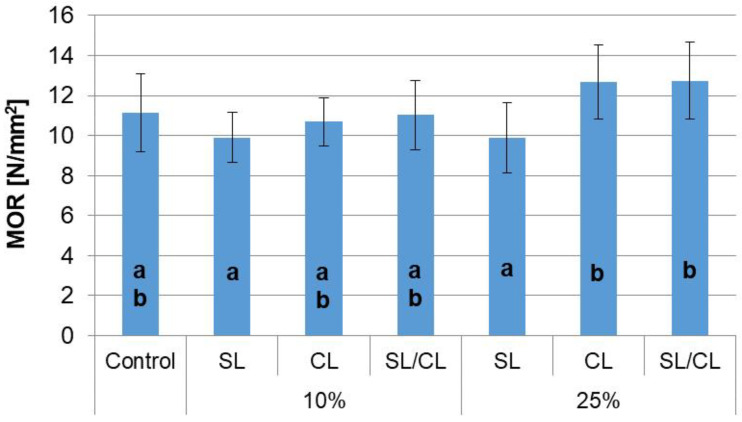
Modulus of rapture of particleboards. a,b—homogenous group by Tukey test.

**Figure 5:**
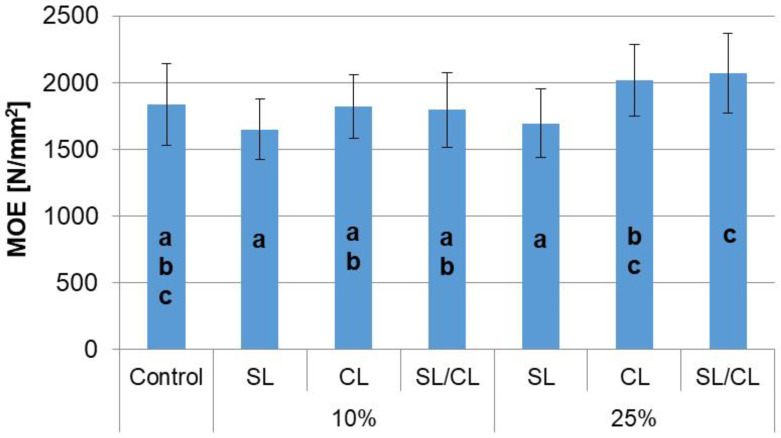
Modulus of elasticity of particleboards. a,b,c—homogenous group by Tukey test.

In the case of the values of the static bending strength (MOR) and the modulus of elasticity (MOE), it can be concluded that in general, the addition of hemp shives to the face layers of the particleboards decreases the value of these parameters compared to conventional boards (Figure 4 and Figure 5). In contrast, the addition of hemp shives to the core layer generally increases the MOR and MOE values compared to the MOR and MOE values observed for conventional panels. However, it should be noted that the observed differences in the value of MOR and MOE of particleboards with a 10% or 25% share of hemp shives compared to particleboards made entirely of industrial wood particles are not statistically significant.

The observed relationships are confirmed by the results of the research presented by Moulan [23], who showed that the increase in the share of hemp shives in the core layer improves its MOR and MOE. Also, Li et al. [42] show that the increase in the share of hemp shives in a single-layer particleboard increases the bending strength and modulus of elasticity.

The analysis of variance showed that the layer with the hemp shives share was a statistically significant factor influencing the value of static bending strength and modulus of elasticity (Table 4). The contribution percentage for this factor amounted to 21% for MOR and 16% for MOE (Table 4), while the shares of hemp shives at the levels of 10% and 25% had a slight impact on the MOR and MOE, which is confirmed by a contribution percentage below 10%. However, it should be noted that the contribution percentage for the error was as high as 63% in the case of MOR and 71% for MOE, which may indicate that the discussed parameters are influenced by factors not included in this study.

**Figure 6:**
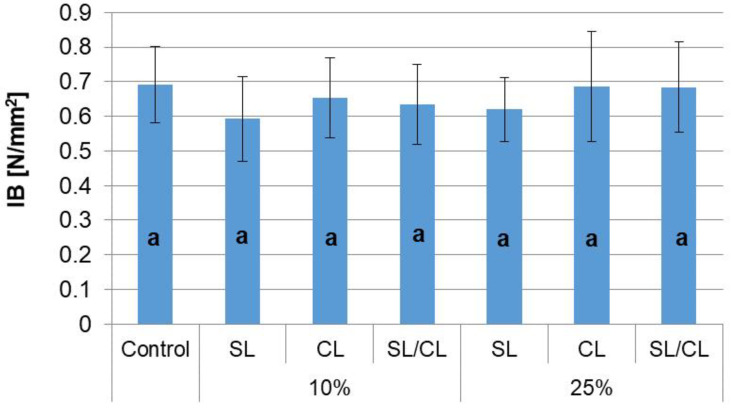
Internal bond of particleboards. a—homogenous group by Tukey test.

The determination of the tensile strength (IB) showed no statistically significant differences between the conventional particleboards and particleboards with a share of hemp shives (Figure 6). Moreover, the analysis of variance showed that both the levels of hemp shives shares and the layer to which hemp shives are added are statistically insignificant factors (Table 4). It should be noted that the obtained results do not confirm the relationships observed by Moulan [23] and Li et al. [42], who showed that the value of IB increases with the content of hemp shives. However, this relationship could not be observed due to the low 25% share of hemp shives, while the authors mentioned investigated particleboards even entirely made of hemp shives.

**Figure 7:**
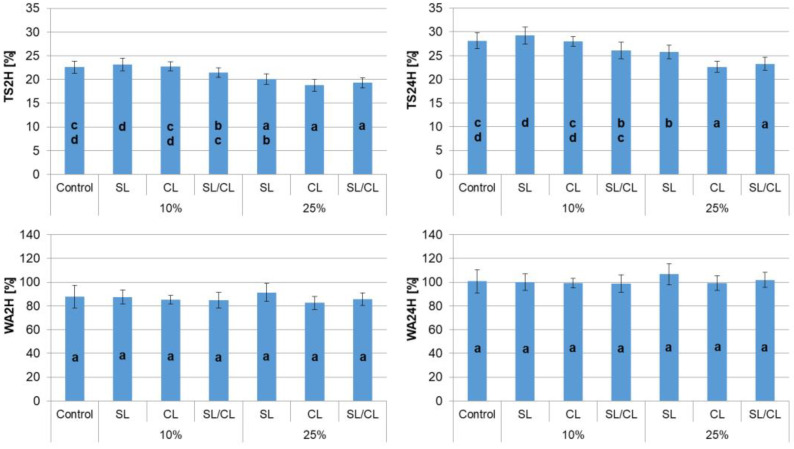
Thickness swelling and water absorption after 2 and 24 h of soaking in water. a,b,c,d—homogenous group by Tukey test.

In the case of thickness swelling (TS) after soaking in water for 2 and 24 h, a decrease in the TS value was observed for particleboards with a 25% share of hemp shives compared to the particleboards made entirely of industrial wood particles (Figure 7). The highest decrease in swelling was noticeable for particleboards with hemp shives in the core layer or the surface and core layers. Interestingly, the share of hemp shives in the range of 10–25% does not affect the water absorption value of the tested particleboards, regardless of the variant of the manufactured particleboards.

Thickness swelling is one of the important parameters of the particleboards produced, as it determines their resistance to moisture. It should be noted that the TS value may be influenced by many factors, such as the raw material used for production, board density, degree of gluing, hydrophobic substances and pressing parameters [40,43]. In the case of the tested particleboards, both the share of hemp shives and the layer to which hemp shives are added turned out to be statistically significant factors influencing TS (Table 5).

## 4. Conclusions

Based on the research, it can be concluded that hemp shives can be used as a 10% and 25% substitute for raw wood material in the production of particleboards. The 25% addition to the core layer or to the surface and core layers allowed us to obtain the highest strength parameters (MOR and MOE) of the manufactured particleboards. It seems that particleboards with the best physical and mechanical properties have hemp shives added to the core layer or to the top and core layer but not only in the surface layers. Moreover, the share of hemp shives in particleboards has a positive effect on the moisture resistance of the manufactured particleboards, reducing their thickness swelling, which is a common problem when using alternative lignocellulosic raw materials. It should also be noted that the 10% and 25% shares of hemp shives in the particleboards do not statistically affect high IB value (all above the minimum requirements of EN 312 even for heavy load-bearing panels) and water absorption. This research points in the direction of using waste materials in the form of crop biomass in particleboard production, an inevitable alternative to wood resources.

## Figures and Tables

**Table 1:** The fractional compositions of the particles used for the core layer and surface layers.

Fraction, mm	Content of the Fraction, %			
	Industrial Wood Particles		Hemp Shives	
	Core Layer	Surface Layers	Core Layer	Surface Layers
6	13.0	–	6.1	–
4	19.0	–	19.4	–
2	51.0	0.6	42.2	14.2
1.25	13.0	14.3	15.2	45.2
0.63	3.6	55.5	7.6	18.3
0.49	0.2	11.4	5.4	12.2
0.385	0.1	7.8	2.6	9.7
dust	0.1	10.4	1.5	0.4

**Table 2:** Variants of manufactured boards.

Variant	Core Layer	Surface Layers	Share of Hemp Shives (% of Dry Mass)
A	-	-	0
B	-	x	10
C	-	x	25
D	x	-	10
E	x	-	25
F	x	x	10
G	x	x	25

**Table 3:** The average density of manufactured particleboards.

		Variant						
		A	B	C	D	E	F	G
Density, kg/m^3^	Average	651 a	639 a	646 a	645 a	641 a	649 a	634 a
	St. dev	39	35	30	34	38	37	39

a—homogenous group by Tukey test.

**Table 4:** Statistical significance of factor influence on mechanical properties of particleboards.

	MOR		MOE		IB	
	*p*	Pc (%)	*p*	Pc (%)	*p*	Pc (%)
share	0.005	9.88	0.013	8.72	0.286	2.19
layer	0.000	21.23	0.003	16.89	0.264	5.15
share × layer	0.116	5.29	0.369	2.69	0.959	0.16
error		63.60		71.70		92.50

*p*—probability of error; Pc—percentage of contribution.

**Table 5:** Statistical significance of factor influence on physical properties of particleboards.

	TS2H		TS24H		WA2H		WA24H	
	*p*	Pc (%)	*p*	Pc (%)	*p*	Pc (%)	*p*	Pc (%)
share	0.000	59.54	0.000	50.81	0.649	0.34	0.085	5.22
layer	0.004	6.74	0.000	19.75	0.018	14.37	0.152	6.60
share × layer	0.044	3.67	0.026	3.91	0.263	4.52	0.340	3.73
error		30.04		25.52		80.77		84.45

*p*—probability of error; Pc—percentage of contribution.

## Data Availability

Not applicable.
